# Supplementary material for: Assessing trends and vulnerabilities in the mutualism between whitebark pine (Pinus albicaulis) and Clark’s nutcracker (Nucifraga columbiana) in national parks of the Sierra-Cascade region
Source: PLoS One. 2020 Oct 14;15(10):e0227161. doi: 10.1371/journal.pone.0227161 (PMC7556478; doi:10.1371/journal.pone.0227161)

**S1 File. Data exploration.**

Appendix. Ray C, Rochefort RM, Ransom JI, Nesmith JCB, Haultain SA, Schaming TD, Boetsch JR, Holmgren AL, Wilkerson RL, Siegel RB. Assessing trends and vulnerabilities in the mutualism between whitebark pine (*Pinus albicaulis*) and Clark’s nutcracker (*Nucifraga columbiana*) in national parks of the Sierra-Cascade region. PLoS ONE.

**Interannual variation in covariates of Clark’s nutcracker detection versus non-detection.** Point-count station characteristics were differentiated according to whether Clark’s nutcracker (CLNU) was detected or not, using generalized additive models (GAMs) to represent interannual variation in mean covariate values at detection sites (red lines) versus non-detection sites (blue dashed lines) and their 95% confidence intervals (shaded regions). Data were drawn from 2005-2016 surveys in Mount Rainier National Park (MORA) and North Cascades National Park Service Complex (NOCA). Penalized maximum likelihood was used to avoid over-fitting each time series (Wood et al. 2016), resulting in a variety of trend forms, from linear to highly complex. We would expect time-series of detection and non-detection sites to overlap for covariates that do little to explain nutcracker detection or presence. Similarly, divergence or convergence between trends might indicates covariate relationships that are changing through time due to shifting controls on the distribution of CLNU in these parks.

The potential for effects of *elevation* and *dense cover* on nutcracker abundance was evident from GAM fits to nutcracker detection data. Nutcracker detections were concentrated at the highest elevations in each park (Fig S1.2 a-b), although detection elevations tended to decline toward the end of the study period. Nutcrackers were more commonly detected in areas with less *dense cover* (Fig S1.2 c-d), especially in MORA. Aside from *elevation* and *dense cover*, GAMs suggested relatively minor differentiation between time-series of occupied and unoccupied point-count stations for the other candidate predictors of nutcracker detection and abundance considered here (*hour*, *noise*, *slope*, *aspect*, *forest* cover, *MST* and *PAS*; S1.1-S1.4). However, certain time series suggested complex dynamics over time and striking distinctions between parks. For example, nutcrackers in MORA and NOCA differed in their use of both *slope* and *aspect* for much of the study period (S1.3 a-d). Nutcrackers in NOCA used steeper slopes on more northern aspects and displayed variable but opposite patterns in their use of forested areas; in years when nutcracker use of *forest* areas was highest in MORA, it was often lowest in NOCA, and conversely (Fig S1.2 e-f). Detection and non-detection sites did not differ appreciably in lagged or non-lagged *MST* or *PAS* (S1.3-S1.4), but isolated climatic events might have had pronounced effects. For example, nutcrackers were detected only at point-count stations with relatively low *PAS* in 2008 and 2009, following the anomalously high *PAS* event of 2008 in NOCA.

Reference

Wood SN, Pya N, Saefken B. Smoothing parameter and model selection for general smooth models (with discussion). J Am Statistical Assoc. 2016; 111: 1548-1575. doi: 10.1080/01621459.2016.1180986.

*Figures begin on next page.*

Figure S1.1. Interannual variation in covariates of Clark’s nutcracker (CLNU) detection (solid curves) and non-detection (dashed curves) at avian point-count stations in Mount Rainier National Park (MORA) and North Cascades National Park Service Complex (NOCA). Detections sometimes occurred during surveys conducted later in the year in MORA (a) and conversely in NOCA (b), and detection dates were often out of sync between parks, but there was no consistent relationship between date and CLNU detections. Strong overlap between curves in (c) and (d) suggests CLNU detection was not related to the daily timing of counts. Partial overlap between curves in (e) and (f) suggests CLNU detection was sometimes related to ambient noise or noise correlates. CLNU were not detected in MORA in 2016.


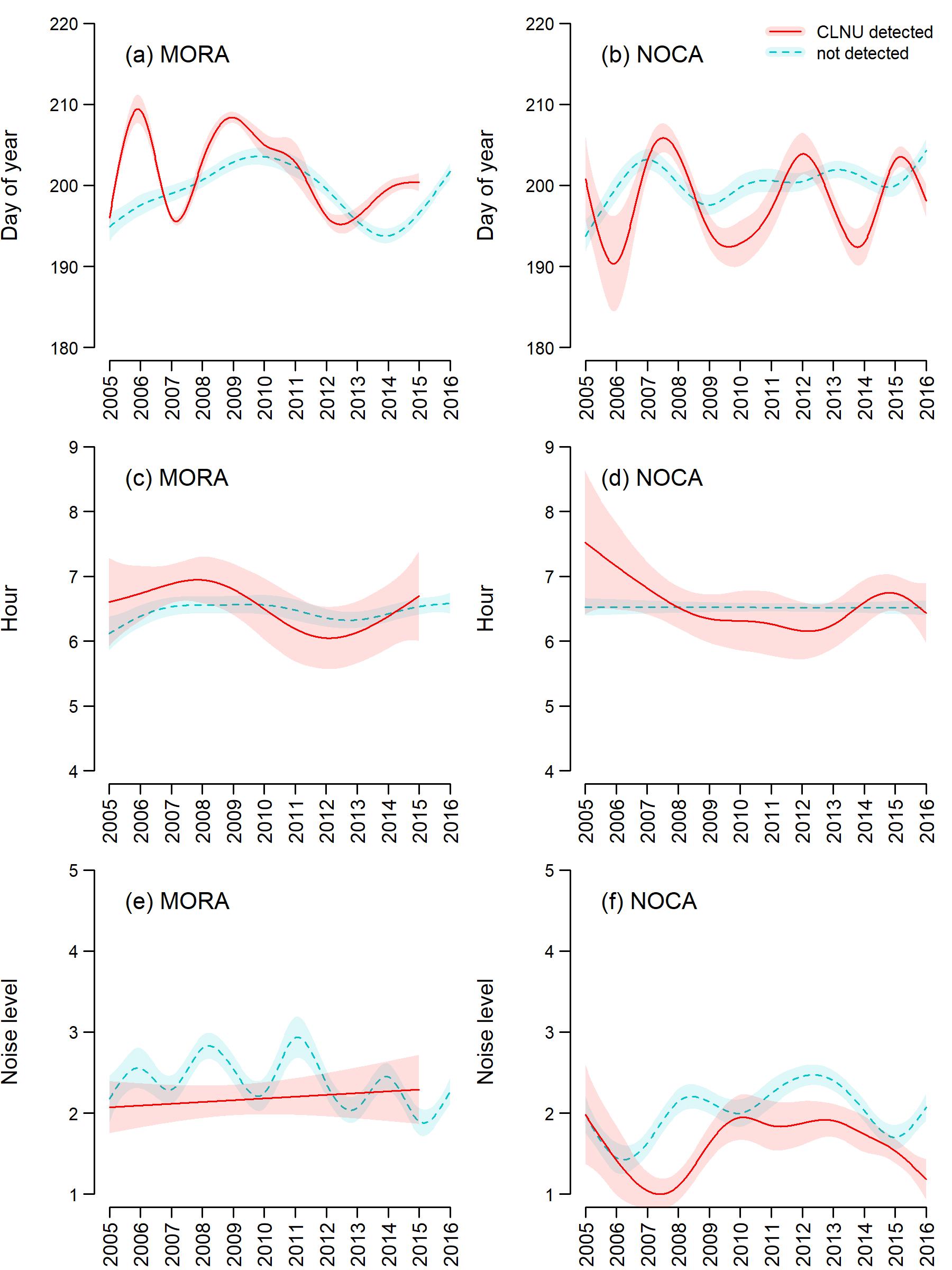


Figure S1.2. Interannual variation in covariates of Clark’s nutcracker (CLNU) detection (solid curves) and non-detection (dashed curves) at avian point-count stations in Mount Rainier National Park (MORA) and North Cascades National Park Service Complex (NOCA). In both parks, detections consistently occurred at much higher elevations (a-b) and where vegetative cover was not dense (c-d), suggesting that CLNU are harder to detect under dense cover and/or prefer more open and/or higher elevation habitats. Detections also tended to be in less forested habitat in each park (e-f), but this pattern was highly variable and somewhat out of sync between parks. CLNU were not detected in MORA in 2016.
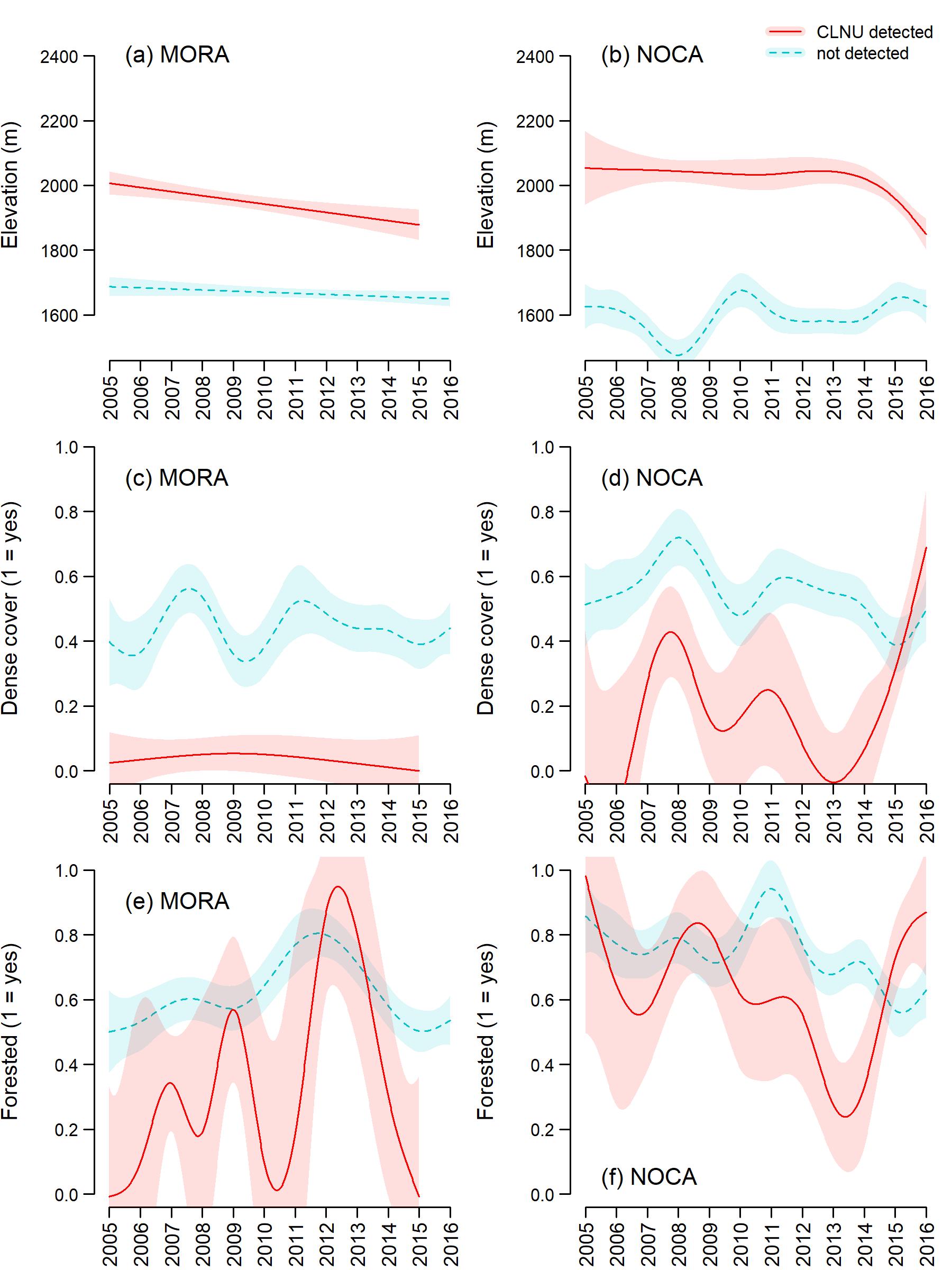


Figure S1.3. Interannual variation in covariates of Clark’s nutcracker (CLNU) detection (solid curves) and non-detection (dashed curves) at avian point-count stations in Mount Rainier National Park (MORA) and North Cascades National Park Service Complex (NOCA). In both parks, strong overlap between curves suggests that detection and non-detection sites did not differ in slope (a-b). Detection sites often differed in aspect, however, with CLNU detected on more south-facing slopes in MORA (c) and on more north-facing slopes in NOCA (d). The mean spring temperature (MST) anomaly (measured as MST in year *t* minus 1971-2000 average MST) was nearly identical between detection and non-detection sites, despite being quite variable over 2005-2016 (e-f). CLNU were not detected in MORA in 2016.


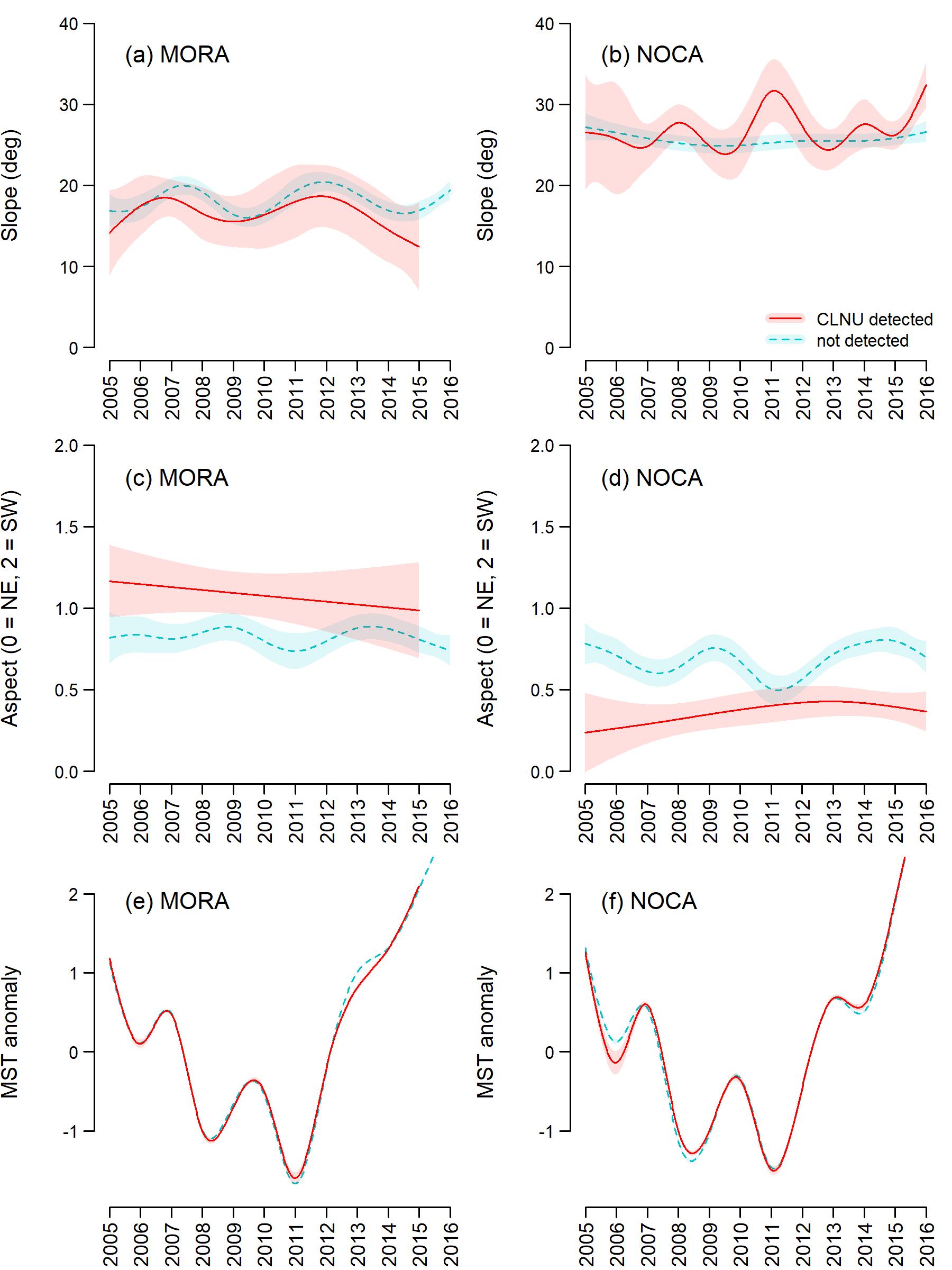


Figure S1.4. Interannual variation in covariates of Clark’s nutcracker (CLNU) detection (solid curves) and non-detection (dashed curves) at avian point-count stations in Mount Rainier National Park (MORA) and North Cascades National Park Service Complex (NOCA). Detection and non-detection sites were generally similar in precipitation-as-snow (PAS) of the current year (August *t*-1 through July *t*), depicted here (a-b) as an anomaly (PAS in year *t* minus 1971-2000 average PAS). Isolated anomalies might have had pronounced effects; see 2013 (a) and 2006 (b). Detection and non-detection sites were also similar in mean spring temperature (MST) of the previous year, depicted here (c-d) as a lag-1 anomaly (MST in year *t*-1 minus 1971-2000 average MST). Finally, detection in year *t* was not consistently distinguished by the PAS anomaly of the previous year (e-f): PAS differed by detection class only in NOCA during a strong anomaly in 2006. CLNU were not detected in MORA in 2016.
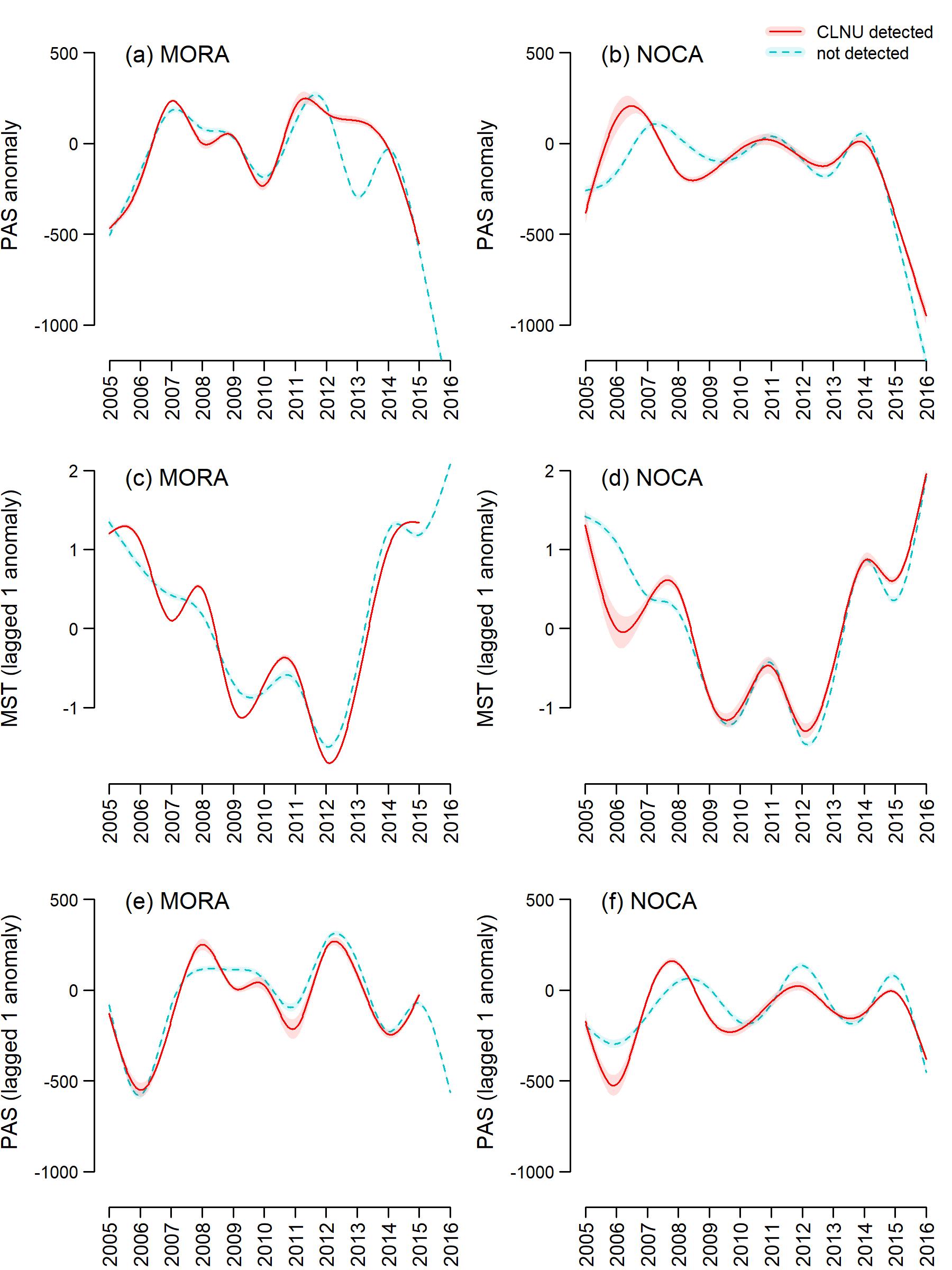

Supplement: S1 File — (DOCX) [file pone.0227161.s001.docx]
