# Supplementary material for: Assessing trends and vulnerabilities in the mutualism between whitebark pine (Pinus albicaulis) and Clark’s nutcracker (Nucifraga columbiana) in national parks of the Sierra-Cascade region
Source: PLoS One. 2020 Oct 14;15(10):e0227161. doi: 10.1371/journal.pone.0227161 (PMC7556478; doi:10.1371/journal.pone.0227161)
Supplement: S2 File — (DOCX) [file pone.0227161.s002.docx]

**S2 File. Model development.**

Appendix. Ray C, Rochefort RM, Ransom JI, Nesmith JCB, Haultain SA, Schaming TD, Boetsch JR, Holmgren AL, Wilkerson RL, Siegel RB. Assessing trends and vulnerabilities in the mutualism between whitebark pine (*Pinus albicaulis*) and Clark’s nutcracker (*Nucifraga columbiana*) in national parks of the Sierra-Cascade region. PLoS ONE.

**Preliminary analyses to identify adequate models for exploration of focal hypotheses.** Our goal was to compare models with and without effects of whitebark pine on Clark’s nutcracker, using a hierarchical mixed model linking four generalized linear models, each with multiple potential covariates. Linear predictors for each sub-model appear below and are detailed in the main text. The ***x*** in each equation represents a vector of candidate covariates that might explain important sources of variation in the four response variables, which include *q* (per-minute probability of nutcracker non-detection), *σ* (scale of the half-normal nutcracker detection distance distribution), *λ_N_* (expected nutcracker abundance) and *λ_W_* (expected whitebark seed production). Important features of this model system are (i) equations (1) and (2) together inform the observation model for nutcrackers; (ii) latent nutcracker abundance = *N_kt_* ~ Poisson(*λ_Nkt_*); and (iii) ***T*** in equation (3) can take the value of *year* or *W_t_*, where *W_t_* is a proxy for tree seed production. In the example below, *W_t_* is based on tree counts and is modeled as *W_kt_* ~ Poisson(*λ_Wkt_*) in equation (4).

logit(*q_kt_*) = *β_q0_* + ***β****_q_****x****_qkt_* (1)

log(*σ_kt_*) = log(*σ_0_*) + ***β****_σ_****x****_σkt_* (2)

log(*λ_Nkt_*) = *β_N0_* + ***β****_N._****T*** + ***β****_N._****x****_Nkt_* + *ε_kt_* + *year_t_* + *transect_k_* (3)

log(*λ_Wkt_*) = *β_W0_* + *β_W._year_t_* + ***β****_W._****x****_Wkt_* + *stand_k_* + *plot_k_* (4)

We used data exploration (S1 File) to suggest key covariates of our observation and population processes, and applied a two-step procedure for building a model ‘adequate’ for describing these processes in the absence of a seed proxy effect on nutcrackers in equation 3. We hoped to include only a few, key covariates in this system in order to facilitate convergence of parameter estimates, avoid time spent estimating posterior densities for extra parameters, and avoid over-fitting our data before introducing seed proxy effects. We defined ‘adequate’ in terms of Bayesian *P*-values associated with posterior predictive checks, where 0.2 < *P* < 0.8 indicated adequate fit, and we removed covariates in a backward stepwise process while fit remained adequate. Our two-step procedure involved covariate selection for the observation process followed by the population process, as follows:

1. Observation model development began by filling equations (1) and (2) with candidate covariates (Table 4), while avoiding high correlation (Pearson’s *ρ* or Kendall’s τ > 0.5) among covariates in the same model. Candidate covariates of the observation process were not highly correlated in any park, but *noise* could affect either *q* or *σ*, so we considered one model in which ***x****_q_ =* {*day*, *hour*, *noise*} while ***x****_σ_ =* {*observer*, *dense cover*}, and a separate model in which ***x****_q_ =* {*day*, *hour*} while ***x****_σ_ =* {*observer*, *noise*, *dense cover*}. In each model, ***T*** and ***x****_N_* in equation (3) were set to 0, which also eliminated *W_k_* or *W_t_* and equation (4) from the model. We then fit each of these relatively full models using JAGS (S3 File) called from R, and reviewed the 95% credible intervals for each fitted effect (*β*) as well as Bayesian *P*-values for posterior predictive checks on *p_a_* and *p_d_*. The spatial models for YOSE and for SEKI as well as the temporal model for MORA remained adequate when all covariates had been removed from equations (1) and (2). The temporal model for NOCA was not adequate unless we retained an observer effect on *σ*. Random year effects were not supported or included in adequate models for MORA, and were not included in spatial models.
2. Beginning with the covariates selected in step (i), equation (3) was then filled with candidate covariates (Table 4) while avoiding high correlation among covariates in the same model by constructing several different models: 1) ***x****_N_ =* {*elevation*, *aspect*, *slope*}, 2) ***x****_N_ =* {*forest, PAS*, *rMST*}, and 3) ***x****_N_ =* {*dense cover*, *PAS*, *rMST*}. In each model, ***T*** was again set to 0 in order to evaluate covariates in absence of the focal process. We then followed the procedure in (i) to identify a simple, adequate model for each park. The spatial models for YOSE and SEKI were not adequate unless we retained *elevation* as a covariate of *λ_Nkt_*. The temporal model of MORA was not adequate unless we retained an effect of *dense cover* on *λ_Nkt_* and dropped the overdispersion term *ε_kt_*. The temporal model of NOCA was not adequate unless we retained both a random effect of *year* and a fixed effect of *elevation* on *λ_Nkt_*, and dropped the overdispersion term.

The key covariates identified in step (ii) were then used in additional analyses to identify added effects of *W_k_* (spatial models) or *year* and *W_t_* (temporal models), after making the appropriate changes in ***T***. Results of this exploratory analysis should be confirmed through formal model selection as monitoring continues to enlarge the dataset available for analyses. Data used in these analyses are available from the National Park Service Data Store at https://irma.nps.gov/DataStore/Reference/Profile/2277917 (NCCN parks) and https://irma.nps.gov/DataStore/Reference/Profile/2278109 (SIEN parks).
