# Supplementary material for: Assessing trends and vulnerabilities in the mutualism between whitebark pine (Pinus albicaulis) and Clark’s nutcracker (Nucifraga columbiana) in national parks of the Sierra-Cascade region
Source: PLoS One. 2020 Oct 14;15(10):e0227161. doi: 10.1371/journal.pone.0227161 (PMC7556478; doi:10.1371/journal.pone.0227161)
Supplement: S3 File — (DOCX) [file pone.0227161.s003.docx]

**S3 File. Spatial and temporal model scripts.**

# Appendix: Ray C, Rochefort RM, Ransom JI, Nesmith JCB, Haultain SA,

# Schaming TD, Boetsch JR, Holmgren AL, Wilkerson RL, Siegel RB.

# Assessing trends and vulnerabilities in the mutualism between whitebark

# pine (*Pinus albicaulis*) and Clark’s nutcracker (*Nucifraga columbiana*) in

# national parks of the Sierra-Cascade region. PLOS ONE.

# Combined hierarchical distance-sampling and time-removal

# N-mixture model for point-transect counts of Clark's nutcracker (cn)

# regressed on proxies of whitebark pine seed production (wp):

# (i) Spatial model; wp = whitebark pine cover from park veg map

# (ii) Temporal model; wp = live whitebark pine trees with blister rust;

# wp = sampled in some years, modeled in others as rust trees ~ Year

# Contents: R/JAGS code adapted from Amundson et al. (2014)

# for estimation of population trends from point-count data

# using an N-mixture model to fit covariate effects on trend

# and on each of two components of detection probability:

# availability based on time-removal data from intervals varying

# in length (Farnsworth et al. 2002), and perceptibility based

# on detection distance. Adapted for use with Clark's nutcracker

# monitoring data by Ray et al. (2017).

# References:

# Amundson CL, Royle JA, Handel CM. 2014. A hierarchical model combining

# distance sampling and time removal to estimate detection probability

# during avian point counts. The Auk 131:476–494.

# Farnsworth GL, Pollock KH, Nichols JD, Simons TR, Hines JE, Sauer JR.

# 2002. A removal model for estimating detection probabilities from

# point-count surveys. The Auk 119:414–425.

# Ray C, Saracco JF, Holmgren AL, Wilkerson RL, Siegel RB, Jenkins KJ,

# Ransom JI, Happe PJ, Boetsch JR, Huff MH. 2017. Recent stability of

# resident and migratory landbird populations in National Parks of the

# Pacific Northwest. Ecosphere. 8(7):e01902. 10.1002/ecs2.1902.

# (i) Spatial model; wp = whitebark pine cover from park veg map

# VARIABLES

# y = count of birds per point (associated w/surveyid, not tinterval)

# surveyid = survey point/site ID for each detection (individual or group)

# observer = observer during point-year (1 = crew leader; 2 = other)

# dclass = distance class per detection

# tinterval = time interval per detection

# nsurveys = number of potential surveys (number of pt-yrs)

# nobs = number of individual birds (or groups) detected

# delta = distance width for each bin (set as equal intervals here)

# nbreaks = number of distance bins

# mdpts = midpoints of distance bins

# maxd = maximum truncated distance (e.g., 200 m)

# J = number of time periods

# ... = standardized covariates dense, elev, noise, hour, aspect, wp cover

# ntrans = total number of transects

# tran = transect ID for each point

# day = ordinal date of survey

# Year = survey year value (2011:2016 rescaled as 1:6)

# nyears = number of survey years (2011-2016 = 6)

# Nst = initial value for estimate of N - must be close to N

# INDICES

# k in 1:nsurveys # surveys across points and years (pt-yr)

# b in 1:nbreaks # distance bins

# j in 1:J # time intervals

# i in 1:nobs # indiv detections, each having a distance category

# t in 1:ntrans # transects

# PARAMETERS TO ESTIMATE

# musigma = mean scale parameter across sites (half normal here)

# mupdet = mean probability of perceptibility

# mupavail = mean probability of availability

# a0 = intercept for availability

# b.a1, b.a2, ... = coefficients of covars in availability model

# sigma0 = intercept for perceptibility

# b.p1, b.p2, ... = coefficients of covars in perceptibility model

# b1, b2, ... = coefficients of covars in abundance model

# N = mean site-level abundance per survey (point-year)

# mu.tran = mean abundance intercept across transects

# sd.tran = SD of random transect effect

# bayesp.pd = Bayesian p-value for pd model

# bayesp.pa = Bayesian p-value for pa model

# dens.ha = density of birds per hectare = totN/area surveyed

file.name <- outfile.name

cat("

model {

# PRIORS

a0 ~ dnorm(0,0.01) # availability intercept

b.a1 ~ dnorm(0,0.01) # coefs of availability covars

b.a2 ~ dnorm(0,0.01)

sigma0 ~ dunif(0,200) # scale of detection intercept

b.p1 ~ dnorm(0,0.01) # coefs of perceptibility covars

b.p2 ~ dnorm(0,0.01)

b0 ~ dnorm(0,0.01) # abundance intercept

b1 ~ dnorm(0,0.01) # coefs of abundance covars

b2 ~ dnorm(0,0.01)

b3 ~ dnorm(0,0.01)

b4 ~ dnorm(0,0.01)

# random transect effect on abundance intercept

for (t in 1:ntrans) {

traneff[t] ~ dnorm(0,tau.tran)

}

tau.tran <- pow(sd.tran,-2)

sd.tran ~ dunif(0,10)

# random year effect on abundance

for (t in 1:nyears) {

yeareff[t] ~ dnorm(0,tau.year)

}

tau.year <- pow(sd.year,-2)

sd.year ~ dunif(0,10)

for (i in 1:2) { # observer effect

obseff[i] ~ dunif(0,200)

}

# overdispersion

for (k in 1:nsurveys){

od[k] ~ dnorm(0.0,tau.od)

}

tau.od ~ dgamma(0.001,0.001)

sd.od <- pow(tau.od,-0.5)

# DETECTION PROBABILITY FUNCTIONS

for (k in 1:nsurveys) {

# covariates of availability and perceptibility

logit(q[k]) <- a0 #+ b.a1*hour[k]

log(sigma[k]) <-log(sigma0) #+ b.p1*noise[k]

# distance-based estimation of detection probability

for (b in 1:nbreaks) {

log(g[b,k]) <- -mdpts[b]*mdpts[b]/(2*sigma[k]*sigma[k]) # half-normal

f[b,k] <- (2*mdpts[b]*delta[b])/(maxd*maxd) # subscripted delta: bin widths vary

#f[b,k] <- (2*mdpts[b]*delta)/(maxd*maxd)

pi.d[b,k] <- g[b,k]*f[b,k] # p(detection) per pt-bin

pi.d.c[b,k] <- pi.d[b,k]/p.d[k] # conditional form standardized by p(detected)

} # b in 1:nbreaks

p.d[k] <- sum(pi.d[,k]) # p.d is a sum over all dclass bins

# removal-based estimation of availability given unequal intervals

pi.a[1,k] <- 1-pow(q[k],3) # p(available in interval j=1)

pi.a[2,k] <- pow(q[k],3)*(1-pow(q[k],2)) # p(avail in j=2)

pi.a[3,k] <- pow(q[k],5)*(1-pow(q[k],2)) # p(avail in j=3)

# p(available in each interval j | available in at least one interval)

for (j in 1:J) {pi.a.c[j,k] <- pi.a[j,k]/p.a[k]}

p.a[k] <- sum(pi.a[,k]) # p(avail in >=1 interval)

} # k in 1:nsurveys

# OBSERVATION-LEVEL MODEL

for (i in 1:nobs) {

# single binomial trial with categorical dist linking dclass & tinterval to pt

dclass[i] ~ dcat(pi.d.c[,surveyid[i]]) # p(outcome = 1 to nbreaks dclasses)

tinterval[i] ~ dcat(pi.a.c[,surveyid[i]]) # p(outcome = 1 to J intervals)

} # i in 1:nobs

# ABUNDANCE ESTIMATION

for (k in 1:nsurveys) {

# counts as a function of number available and detection probability

y[k] ~ dbin(p.d[k],n.a[k])

# number available for sampling as a function of abundance and p(available)

n.a[k] ~ dbin(p.a[k],N[k])

# abundance model

N[k] ~ dpois(lambda[k]) # predicted abundance per survey (point-year)

# covariates of abundance

log(lambda[k]) <- b0 + b1*elev[k] + od[k] + traneff[tran[k]]

} # k in 1:nsurveys

# GOODNESS OF FIT STATS

for (k in 1:nsurveys) {

n.a.fit[k] ~ dbin(p.a[k],N[k]) # new realization of model

y.fit[k] ~ dbin(p.d[k],n.a[k])

e.pd[k] <- p.d[k]*n.a[k] # original model prediction

E.pd[k] <- pow((y[k]-e.pd[k]),2)/(e.pd[k]+0.5) # dev from obs

E.New.pd[k] <- pow((y.fit[k]-e.pd[k]),2)/(e.pd[k]+0.5) # dev from pred

e.pa[k] <- p.a[k]*N[k]

E.pa[k] <- pow((n.a[k]-e.pa[k]),2)/(e.pa[k]+0.5)

E.New.pa[k] <- pow((n.a.fit[k]-e.pa[k]),2)/(e.pa[k]+0.5)

} # k in 1:nsurveys

fit.pd <- sum(E.pd[]) # dev from obs

fit.new.pd <- sum(E.New.pd[]) # dev from pred

LOF.pd <- fit.pd/fit.new.pd

bayesp.pd <- step(fit.new.pd-fit.pd) # p-value for perceptibility model

fit.pa <- sum(E.pa[])

fit.new.pa <- sum(E.New.pa[])

LOF.pa <- fit.pa/fit.new.pa

bayesp.pa <- step(fit.new.pa-fit.pa) # p-value for availability model

# SUMMARY STATS

mupavail <- mean(p.a[]) # mean probability of availability

mupdet <- mean(p.d[]) # mean probability of perceptibility

musigma <- mean(sigma[]) # mean scale parameter across sites

ha <- maxd*maxd*3.14159/10000 # hectares surveyed at each pt

dens.ha <- mean(N[])/ha # mean point-level density across park

# we want to summarize N by pt-count station, which is cryptic

# in k, so we create a new index, ptyr, to access our data;

# ptyr is a matrix version of k, allowing summaries by year

# (row) or by plot/station (column); ptyr has a row for every

# plot/station and a col for every nyears; each element (pt-yr)

# holds the corresponding index of k; ptyr is defined as follows,

# and passed to the model:

# ptyr <- matrix(0,length(Year)/nyears,nyears)

# for (i in 2011:2016) {ptyr[,(i-2010)] <- which(Year==i)}

# N by pt-ct station (1216 unique stations for YOSE)

for (i in 1:1216) {

N.pt[i] <- sum(N[ptyr[i,]]) # here N is summarized by column

}

}

",file=file.name)

# (ii) Temporal model; wp = live whitebark pine trees with blister rust;

# wp = sampled in some years, modeled in others;

# wp model = rust trees ~ Year...

# VARIABLES - whitebark

# y.wp = wp seed proxy = rust-trees

# yr = Yr = whitebark data year

# stand = stratum or transect; factors 1-8 for MORA, 1-5 for NOCA

# nstands = 8 or 5 as above (like ntrans for nutcracker)

# nsurveys.wp = number of potential surveys (number of plot-yrs)

# ... = standardized covariates elev.wp, slope.wp, aspect.wp

# nyears.wp = number of potential (modeled) tree survey years

# Rst = initial value for estimate of rust-trees (must be close)

# INDICES - whitebark

# k in 1:nsurveys.wp # surveys (348 in MORA)

# t in 1:nstands # transects (8 in MORA)

# p in 1:nplots # plots (29 in MORA)

# PARAMETERS TO ESTIMATE - whitebark

# bw0, bw1, bw2, ... = intercept and coefficients of whitebark covars

# mu.stand = mean abundance intercept across stands

# sd.stand = SD of random stand effect

# bayesp.wp = Bayesian p-value for model

# VARIABLES - nutcracker

# y.cn = count of birds per point (associated w/surveyid, not tinterval)

# surveyid = survey point/site ID for each detection (individual or group)

# observer = observer during point-year

# dclass = distance class per detection

# tinterval = time interval per detection

# nsurveys.cn = number of potential surveys (number of pt-yrs)

# nobs = number of individuals (or groups) detected

# delta = distance width for each bin (set as equal intervals in this example)

# nbreaks = number of distance bins

# mdpts = midpoints of distance bins

# maxd = maximum truncated distance (e.g., 200 m)

# J = number of time periods

# ... = standardized covariates forest, dense, elev, noise, hour...

# ntrans = total number of transects

# tran = transect ID for each point

# day = ordinal date of survey

# Year = survey year value (2005:2016 rescaled as 1:12)

# nyears = number of survey years (2005-2016 = 12)

# Nst = initial value for estimate of N - must be close to N

# INDICES - nutcracker

# k in 1:nsurveys.cn # surveys

# b in 1:nbreaks # distance bins

# j in 1:J # time intervals

# i in 1:nobs # detections (individual or group), each having a distance category

# t in 1:ntrans # transects

# PARAMETERS TO ESTIMATE - nutcracker

# musigma = mean scale parameter across sites (half normal shape in this example)

# mupdet = mean probability of perceptibility

# mupavail = mean probability of availability

# a0 = intercept for availability

# b.a1, b.a2, ... = coefficients of covars in availability model

# sigma0 = intercept for perceptibility

# b.p1, b.p2, ... = coefficients of covars in perceptibility model

# bn1, bn2, ... = coefficients of covars in abundance model

# N = mean site-level abundance per survey (point-year)

# mu.tran = mean abundance intercept across transects

# sd.tran = SD of random transect effect

# bayesp.pd = Bayesian p-value for pd model

# bayesp.pa = Bayesian p-value for pa model

# dens.ha = density of birds per hectare = totN/area surveyed

cat("

model {

# PRIORS whitebark + nutcrackers

bw0 ~ dnorm(0,0.01) # rust-trees intercept

bw1 ~ dnorm(0,0.01) # coefs of whitebark covars

bw2 ~ dnorm(0,0.01)

a0 ~ dnorm(0,0.01) # nutcracker availability intercept

b.a1 ~ dnorm(0,0.01) # coefs of availability covars

b.a2 ~ dnorm(0,0.01)

sigma0 ~ dunif(0,200) # scale of detection intercept

b.p1 ~ dnorm(0,0.01) # coefs of perceptibility covars

b.p2 ~ dnorm(0,0.01)

b0 ~ dnorm(0,0.01) # abundance intercept

b1 ~ dnorm(0,0.01) # coefs of abundance covars

b2 ~ dnorm(0,0.01)

b3 ~ dnorm(0,0.01)

b4 ~ dnorm(0,0.01)

# random stand effect on seed proxy (wp) intercept

for (t in 1:nstands) {

standRE[t] ~ dnorm(0,tau.stand) # I(-16,16)

}

tau.stand <- pow(sd.stand,-2)

sd.stand ~ dunif(0,10)

# fixed stand effects on wp intercept (standFE) and slope (bs)

# useful for modeling the 5-stand study in NOCA

for (l in 1:nstands) {

standFE[l] ~ dnorm(0,0.001)T(-15,15) # intercept bounded to converge

bs[l] ~ dnorm(0,0.001)T(-10,10) # slope

}

# random whitebark plot effect

for (p in 1:nplots) {

plotRE[p] ~ dnorm(0,tau.plot) # I(-16,16)

}

tau.plot <- pow(sd.plot,-2)

sd.plot ~ dunif(0,10)

# random transect effect on nutcracker abundance intercept

for (t in 1:ntrans) {

traneff[t] ~ dnorm(0,tau.tran)

}

tau.tran <- pow(sd.tran,-2)

sd.tran ~ dunif(0,10)

# random year effect on nutcracker abundance

for (t in 1:nyears.cn) {

yeareff[t] ~ dnorm(0,tau.yr.cn)

}

tau.yr.cn <- pow(sd.yr.cn,-2)

sd.yr.cn ~ dunif(0,10)

for (i in 1:2) { # observer effect

obseff[i] ~ dunif(0,200)

}

# overdispersion

for (k in 1:nsurveys){

od[k] ~ dnorm(0.0,tau.od)

}

tau.od ~ dgamma(0.001,0.001)

sd.od <- pow(tau.od,-0.5)

# NUTCRACKER DETECTION PROBABILITY FUNCTIONS

for (k in 1:nsurveys.cn) {

# covariates of availability and perceptibility

logit(q[k]) <- a0 #+ b.a1*hour[k]

log(sigma[k]) <-log(sigma0) #+ b.p1*noise[k]

# distance-based estimation of detection probability

for (b in 1:nbreaks) {

log(g[b,k]) <- -mdpts[b]*mdpts[b]/(2*sigma[k]*sigma[k]) # half-normal

f[b,k] <- (2*mdpts[b]*delta[b])/(maxd*maxd) # subscripted delta if bin widths vary

#f[b,k] <- (2*mdpts[b]*delta)/(maxd*maxd)

pi.d[b,k] <- g[b,k]*f[b,k] # p(detection) per pt-bin

pi.d.c[b,k] <- pi.d[b,k]/p.d[k] # conditional form stdized by p(detected)

} # b in 1:nbreaks

p.d[k] <- sum(pi.d[,k]) # p.d is a sum over all dclass bins

# removal-based estimation of availability given unequal intervals

pi.a[1,k] <- 1-pow(q[k],3) # p(available in interval j=1)

pi.a[2,k] <- pow(q[k],3)*(1-pow(q[k],2)) # p(avail in j=2)

pi.a[3,k] <- pow(q[k],5)*(1-pow(q[k],2)) # p(avail in j=3)

# p(available in each interval j | available in at least one interval)

for (j in 1:J) {pi.a.c[j,k] <- pi.a[j,k]/p.a[k]}

p.a[k] <- sum(pi.a[,k]) # p(avail in >=1 interval)

} # k in 1:nsurveys.cn

# NUTCRACKER OBSERVATION-LEVEL MODEL

for (i in 1:nobs) {

# single binomial trial with categorical dist linking dclass & tinterval to pt

dclass[i] ~ dcat(pi.d.c[,surveyid[i]]) # p(outcome = 1 to nbreaks dclasses)

tinterval[i] ~ dcat(pi.a.c[,surveyid[i]]) # p(outcome = 1 to J intervals)

} # i in 1:nobs

# ABUNDANCE ESTIMATION

# whitebark model: Poisson dist of live rust-trees (rust)

for (k in 1:nsurveys.wp) {

y.wp[k] ~ dpois(rust[k]) # observed rust trees per survey (plot-year)

# covariates of the whitebark metric (W = trees, rust, etc)

log(rust[k]) <- bw0 + bw1*Yr[k] + plotRE[plot.wp[k]]

} # k in 1:nsurveys.wp

# nutcracker model:

for (k in 1:nsurveys.cn) {

# counts as a function of number available and detection probability

y.cn[k] ~ dbin(p.d[k],n.a[k])

# number available for sampling as a function of abundance and p(available)

n.a[k] ~ dbin(p.a[k],N[k])

# abundance model

N[k] ~ dpois(lambda[k]) # predicted abundance per survey (point-year)

# covariates of abundance

log(lambda[k]) <- bn0 + bn1*rust.yr[Year[k]] + bn2*dense[k] + traneff[tran[k]]

} # k in 1:nsurveys.cn

# SUMMARIES AND DERIVED PARAMETERS

# a matrix of points by years is created to hold the corresponding

# indices of k; ptyr is defined as follows, and passed to the model:

# ptyr.wp <- matrix(0,length(Yr)/nyears.wp,nyears.wp)

# for (i in min(Yr):max(Yr)) {ptyr.wp[,(i-min(Yr)+1)] <- which(Yr==i)}

for (i in 1:nyears.wp) {

rust.yr[i] <- mean(rust[ptyr.wp[,i]]) # mean rust-infected trees per year

} # i in 1:nyears.wp

# whitebark: weight by stand/plot unless there's no stand effect on rust/plot

# N by pt-ct station (1216 unique stations for YOSE)

# nutcrackers: there's no stratum effect and in MORA all but one cn was detected

# in the high stratum (above 1350 m), so we can estimate nutcrackers/ha as the

# total number at high stations divided by the (invariant) total number of ha

# represented by our estimate each year, or ha/station (determined by maximum

# detection distance) times the number of stations in the high stratum; maxd

# was 300 m in MORA, 184 m in NOCA, and there were 429 stations at high elev

# in MORA (1012 stns total in MORA and 1181 in NOCA); also, note that in NOCA

# (unlike MORA) distant detections had to be thrown out to achieve independence

# between distance and time to detection, and there were 8 nutcrackers in the

# mid-elev stratum, so we modeled all strata and used all 1181 points surveyed

ha <- maxd*maxd*3.14159/10000*npoints # total ha surveyed

# N/ha/year = cn.ha.yr

for (i in 1:nyears.cn) {

cn.ha.yr[i] <- sum(N[yr.cn[,i]])/ha

}

mupavail <- mean(p.a[]) # mean probability of availability

mupdet <- mean(p.d[]) # mean probability of perceptibility

musigma <- mean(sigma[]) # mean scale parameter across sites

# GOODNESS OF FIT STATS

# whitebark model

for (k in 1:nsurveys.wp) {

y.fit.wp[k] ~ dpois(rust[k]) # create new realization of model

e.wp[k] <- rust[k] # original model prediction

E.wp[k] <- pow((y.wp[k]-e.wp[k]),2)/(e.wp[k]+0.5) # dev from observed

E.New.wp[k] <- pow((y.fit.wp[k]-e.wp[k]),2)/(e.wp[k]+0.5) # dev from predicted

} # k in 1:nsurveys.wp

fit.wp <- sum(E.wp[]) ##dev from obs

fit.new.wp <- sum(E.New.wp[]) ##dev from pred

LOF.wp <- fit.wp/fit.new.wp

bayesp.wp <- step(fit.new.wp-fit.wp) # p-value for model

# nutcracker model

for (k in 1:nsurveys.cn) {

n.a.fit[k] ~ dbin(p.a[k],N[k]) # create new realization of model

y.fit.cn[k] ~ dbin(p.d[k],n.a[k])

N.fit[k] ~ dpois(lambda[k])

e.pa[k] <- p.a[k]*N[k]

E.pa[k] <- pow((n.a[k]-e.pa[k]),2)/(e.pa[k]+0.5)

E.New.pa[k] <- pow((n.a.fit[k]-e.pa[k]),2)/(e.pa[k]+0.5)

e.pd[k] <- p.d[k]*n.a[k]

E.pd[k] <- pow((y.cn[k]-e.pd[k]),2)/(e.pd[k]+0.5)

E.New.pd[k] <- pow((y.fit.cn[k]-e.pd[k]),2)/(e.pd[k]+0.5)

} # k in 1:nsurveys.cn

fit.pd <- sum(E.pd[]) # dev from obs

fit.new.pd <- sum(E.New.pd[]) # dev from pred

LOF.pd <- fit.pd/fit.new.pd

bayesp.pd <- step(fit.new.pd-fit.pd) # p-value for perceptibility model

fit.pa <- sum(E.pa[])

fit.new.pa <- sum(E.New.pa[])

LOF.pa <- fit.pa/fit.new.pa

bayesp.pa <- step(fit.new.pa-fit.pa) # p-value for availability model

}

",file=file.name)
