# Supplementary material for: Assessing trends and vulnerabilities in the mutualism between whitebark pine (Pinus albicaulis) and Clark’s nutcracker (Nucifraga columbiana) in national parks of the Sierra-Cascade region
Source: PLoS One. 2020 Oct 14;15(10):e0227161. doi: 10.1371/journal.pone.0227161 (PMC7556478; doi:10.1371/journal.pone.0227161)
Supplement: Attachment — Submitted filename: plosone_nutcrackerwhitebark_review.docx [file pone.0227161.s004.docx]

Plos one review: Trends in the whitebark pine-Clark’s nutcracker mutualism

Thank you for the opportunity to review this manuscript. It was generally comprehensive and well-written with relevant conclusions to warrant publication in Plos one. I appreciate that the authors attempted to examine multiple facets of potential mutualism and association between nutcracker abundance and whitepine distribution, productivity, and pathogens. The integrated analysis examining the effects of whitepine trends on nutcracker abundance was particularly creative. My one general comment is that the paper is trying to do so much, it is quite difficult to follow through the methods and results. The authors are dealing with two primary analyses (temporal trend and spatial pattern) at 4 different sites, and each analysis has several different response variables and covariate sets. Instead of Figure 2, which provides a schematic of the N-mixture integrated model structure, perhaps a schematic of how each analysis or summary is structured would be much more helpful. Or a table of candidate models for each analysis. Something that structures that information and puts a reference in one place. Also, I suggest using additional sub-headings for different analyses or sites or something that helps readers index what information belongs to what. Other than restructuring the Methods/Results sections to provide additional clarity, the authors could include additional language describing model/covariate selection procedures. Otherwise, my comments are mostly editorial in nature (see below).

Line item comments:

Line 30-31: Remove scientific name from species because its in the abstract?

Line 64: Are names of species originators required as part of Plos one formatting? Suggest omitting here and elsewhere.

Line 90-91: Suggest revising sentence to something more concise – for example, “Whitebark requires Clark’s nutcrackers to disperse its wingless seeds.

Line 111: This objective seems to incorporate only part of your analyses. The following paragraph (lines 117-126) delves more into methods, but doesn’t succinctly state the objective of each analysis – perhaps reword to focus more broadly on study objectives and reserve methodological discussion to the Methods or Discussion sections.

Line 176: Subheading for avian data?

Line 178: Replace ‘to’ with ‘in’?

Line 194-195: I’m not clear on what ‘cover density’ represents. I suggest briefly defining here and perhaps changing lower and higher cover to lower and higher density in parentheses?

Line 211-220: Your spatial analyses and resulting figures seem a bit clunky/rudimentary. I’m confused why you reduced avian count data to apparent presence/absence despite having counts and information to estimate detection probability. If you used those data, you could create a nutcracker density map across SIEN sites that included whitebark or foxtail DBH, # of cone-producing trees, elevation, etc. as covariates. Summarized in grid cells that approximated the size of the area surveyed per point count. From this information you could provide helpful summary statistics such as XX% of the nutcracker population in SIEN occurred in whitebark dominated habitat, while XX% occurred in foxtail habitat. Or, cells with trees > XX DBH had XX% greater density of nutcrackers. Something more tangible than multiple pie charts over landcover layers that are difficult to interpret.

Lines 221-229: Did these avian exploratory analyses actually feed into any other models? It doesn’t appear so – I would delete this section and the corresponding section in the Results – find a way to incorporate the necessary covariates into a single temporal analysis.

Line 231: Can you be more specific than whitebark dynamics? It would add clarity to define the response variables in the GLMs that fed into the N-mixture model as covariates.

Table 3: Not very informative – W = whitebark metrics and x = other covariates. See general comment, but some detailed summary of what actually was examined in each model component would be useful here and for other analyses.

Lines 297-300: I’m confused as to how you actually conducted model selection. Here you state you included each voariate in a set of candidate models with 0-4 additional covariates. Then on lines 323-325 you state you examined GOF statistics and overlapping 95% CIs, but if you included a varying number of covariates per model, you must have somehow decided which covariate combination was the ‘winner’ in order to interpret coefficients? I’m not sure if this was poorly done or I’m missing the authors’ intent. In the Results section, Bayes-P and LOF ratio are reported as if they provide evidence in support of a specific covariate set – which is an inappropriate use of those metrics. Regardless, if you have a candidate model set for each analysis, it would be worth including it as a table or appendix. Further, if you’re looking for a more formal way to conduct Bayesian model selection, there are few options including WAIC or LOO, which can be implemented pretty easily in R using the LOO package by exporting the log likelihood estimates from your jags model and running a couple lines of code. From that you can obtain an information criterion metric that could be used to formally compare models. Regardless, this section needs to be rewritten to facilitate understanding of the actual model/covariate selection process.

Line 316: Why mean = 1.0 here and not 0.0, which is more typical for uninformative random effect priors?

Line 385-388: So you’re really basing your temporal trends on three data points for whitebark metrics. Is the implication of such a minimal sample size on your ability to make inference warranted in the Discussion?

Line 456-473: So, these results are from a series of ad hoc GAMs on the apparent presence/absence of nutcrackers? Why abandon the results of your N-mixture model to delve into covariate effects from this analysis? Could these covariates not have entered into your N-mixture model, and if they weren’t supported, shouldn’t be discussed further?

Line 482: The Discussion is quite long and focuses extensively on scenarios that call their study into question such as dynamics outside of park boundaries and evidence suggesting nutcrackers aren’t that dependent upon whitebark. I think these sections are important to include but could be shortened significantly. Focus on the interesting results from your study, how they fit in the broader literature, what issues occurred with the data and your ability to make inference, and what could be done going forward.

Line 624: All other citations are numbers, why author and year here?
