# Supplementary material for: Assessing trends and vulnerabilities in the mutualism between whitebark pine (Pinus albicaulis) and Clark’s nutcracker (Nucifraga columbiana) in national parks of the Sierra-Cascade region
Source: PLoS One. 2020 Oct 14;15(10):e0227161. doi: 10.1371/journal.pone.0227161 (PMC7556478; doi:10.1371/journal.pone.0227161)
Supplement: Attachment — Submitted filename: ResponseToReviewsPONE-20200809.docx [file pone.0227161.s006.docx]

PONE-D-19-34230R1
Assessing trends and vulnerabilities in the mutualism between whitebark pine (*Pinus albicaulis*) and Clark’s nutcracker (*Nucifraga columbiana*) in national parks of the Sierra-Cascade region
PLOS ONE

RESPONSE TO REVIEW (see RESPONSEs below)

Thank you for such an extensive [revision] of the previous version of the manuscript. I have found the material and methods clearer and more easy to follow. Now, I could understand the sampling protocol as well as the analyses performed and they are correct. All the issues raised by the first referee were solved and he thinks (as I do) that the contribution of this work is relevant.

 However, even though the methods section has been clarified I agree with the second referee that some parts are still difficult to follow.  I had to read Amudson’s work (2014) to fully understand fig. 2. A full formulation of the model will help readers to better understand your work.

 In the current version it is difficult to connect all parts of the model.  For instance, eq 1 reflects the relationship between the probability of non-detection (q) and covariates, but the relationship between (q) and availability is not formulated. Similarly, in eq. 2 the relationship between the scale parameter of the half-normal distribution is stated, but the information about its relationship with perceptibility is scattered throughout the text (see comments below). Most importantly, the link between population parameters and observation processes was not totally connected even though its formulation would be quite simple. (1) A binomial distribution that links the number of individuals observed (y) to those available (n) with a probability of detection (pd). Pd, in turns depends on the effects of covariates on the scale parameter sigma. (2) A binomial distribution that connects the number of available individuals (n) with the actual number of individuals (N) with a probability of availability (pa). Pa depends on the probability of non-detection (q) which is modulated by certain covariates. (3) A poisson model that links the expected number of individuals (lambda) with environmental covariates. Stating these formulas in the text, together with fig. 2 would really help to understand your modeling approach.

RESPONSE: Thank you for the favorable review and detailed suggestion for revision. We have implemented this particular revision within the first paragraph of Methods / Analyses.

In sum, I congratulate the authors for the hard work and the revision of the previous version. I agree with the analyses performed but prior to publication the formulation of the model needs to be within the manuscript (if not in the main text in a supplementary material).  To make the reading more straightforward, I suggest to include the formulation in the main text while shortening some parts (i.e. L265-269).

RESPONSE: We have included text on the model formulation in the main text and have shortened the indicated section as suggested.

Minor comments

L105-120 this part gets a little bit to methodological in the introduction. I think an important contribution of this work is that the modeling approach allows to unify different monitoring protocols. Maybe some of the methodological parts can be summarized and your novel approach can be highlighted with references to other modeling efforts in the same line. This is only a suggestion

RESPONSE: To reduce the methodological detail in this paragraph, we moved one sentence (with details on the dates of survey establishment in each network) into a later paragraph. We opted to retain most other elements of this paragraph in order to support our argument that a unifying model is appropriate and necessary.

Table 1 heading. Please specify that these are NCNN parks and that this dataset will be used for the spatial analyses. For readers that are not familiar with the area there are many acronyms and it gets difficult to follow. The same applies for table 2.

RESPONSE: We made the requested changes to captions of Tables 1 and 2.

L189 How many panels?

RESPONSE: We inserted the number of panels (six).

L204. Please briefly describe aspect for non-specialized readers.

RESPONSE: We have added text to provide guidance for readers.

L207-216 The way climatic covariables are explained is not clear. At the end of this paragraph I was not sure which variables were used. It was not until table 4 that I found out that it was PAS and MSTr. I also got lost with the time lag used (previous or current year) and how an anomaly was defined. I guess it is the MST residual. Please specify this and give a biological interpretation of these residuals. In sum, rewrite this paragraph making sure that the reader knows which climatic variables were used, their interpretation and time-lag .

RESPONSE: We have added text to explain the biological justification for the time-lag we considered. We have also clarified why we used residuals of MST on PAS in our models, citing a paper for this approach, which is adopted simply to avoid collinearity due to correlation between MST and PAS.

L255 Figure 2 legend and along the text.  To make the formulation consistent with that of Amudsons’ I suggest to use the probability of detection instead (ak in Amudsons’ formulation).

RESPONSE: We use *q*, the per-minute probability of non-detection, because *q* was referenced in both the original model of availability by Farnsworth et al. (2002) and by Amundson et al. (2014; e.g., page 480), and because the code we have previously published (Ray et al. 2017) is based on *q*. For these reasons, we are requesting permission to retain *q* as the variable of interest, but we have attempted to explain q more clearly by adding “*q* = 1-*a*” in this clause.

L273 This would need a connection between the half-normal function and the probability of detection. I think that formulation would help. If not, information provided in L273 should be in L291-293 to make it easier to follow.

RESPONSE: We have revised this text as “We then used the common approach of approximating the decline in detection probability with distance from the observer using a half-normal distribution fitted to *b*, our (binned) data on distance-to-detection. Using this approach, *p_d_* was a function of *σ*, the fitted scale parameter of the half-normal distribution [39, 51, 59].”

Table 5 In the spatial analyses were whitebark and foxtail parameters normalized? If I understood correctly in the temporal analyses they were not (because they were estimated). Please clarify this to make sure that comparisons between parameters strength are correct.

RESPONSE: Yes, whitebark and foxtail cover were standardized in the spatial analysis. We have now clarified this point. In the temporal analysis, which used data from NCCN parks, there were no foxtail present, so we did not compare relative effects of whitebark and foxtail on nutcracker dynamics.

Fig. 1B I could not see the dots. I guess they are lines.

RESPONSE: Yes, the dots representing individual point-count stations appear to merge into lines along each transect, so we have revised the text to better describe the figure. Thank you for your attention to detail.

Reviewers' comments:

Reviewer's Responses to Questions

**Comments to the Author**

1. If the authors have adequately addressed your comments raised in a previous round of review and you feel that this manuscript is now acceptable for publication, you may indicate that here to bypass the “Comments to the Author” section, enter your conflict of interest statement in the “Confidential to Editor” section, and submit your "Accept" recommendation.

Reviewer #1: All comments have been addressed

Reviewer #1: The authors have done a great job revising the manuscript following extensive comments by the reviewers and editors. To me, the most interesting finding is that YEAR is the strongest predictor of the observed decline in CLNU detections, strongly suggesting that in addition to declines in food species, other factors may contribute to population dynamics in this species. Overall, this is an important contribution and I look forward to seeing future results from this modeling approach

RESPONSE: Thank you for your favorable review. We have emphasized the effect of year as suggested, especially in the 5^th^ paragraph of the discussion.

Reviewer #3: Thanks for the opportunity to review this paper. The subject matter is interesting, and the introduction and discussion were generally clear, interesting, and well written. The methods, and subsequently results however need substantial work. Following this, the work should be a valuable contribution to the scientific literature.

The main issue is that the methods do not clearly explain exactly what models were fit and how. I believe that there are two overarching problems.

The first is linguistic: The authors fit hierarchical models, which contain many parts. In the description of these parts, the authors often refers to these parts (which are generally link functions or sub-models) as "models" or "GLMs". This is incorrect and very confusing.

As far as I can tell (and this relates to the following problem) the models were fit as a hierarchical model, e.g. JAGS models supp 2. The frequent reference to parts of these hierarchical models as "models" themselves is very confusing. E.g. lines 400-430.

The equations showing the link functions 1, 2, and 3, on lines 433, 437, and 452 are equations, and should be labelled as such. However these are later referred to in text as "models" (lines 462, 468).

Please clarify this section.

RESPONSE: We thank the reviewer for his fair assessment that our terms might prove confusing. We have replaced several instances of “model” with “sub-model” and have added descriptive adjectives (e.g., “observation sub-model”) where necessary. However, we note that many papers on hierarchical models have referred to these sub-models as models and as GLMs. For example, Amundsen et al. (2014) state, “Etterson et al. (2009) created a similar hierarchical N-mixture model using Farnsworth et al.’s (2002) time-removal method to estimate abundance adjusted for p*_a_*. In these models, covariate effects on both abundance and detection can be modeled directly as a generalized linear model.”

The second problem is that I cannot understand how many models were fit and which they were. This is compounded by the first problem which throws the word 'model' around when it should not be.

Tables 5 and 6 show a series of models. I don't understand how you used backwards selection from a full model to arrive at different these different models - shouldn't you arrive at a single model? Further (t5) I don't understand how you eliminated various versions of Wk (t3) to choose whitebark cover as the measure. It may well be explained, but it's not clear to me where from careful reading of the methods and supps. This must be clarified.

In addition to working on your explanation, I suggest three key structural things would go a long way to helping resolve this:

Firstly make a full description of your model specifications as equations. At present only parts of the model are shown, i.e., in equations 1-3. Put the whole model down, either in text or supp. The JAGS code and DAG are only useful for people who already know JAGS and are used to interpreting DAGs. E.g.

N ~ Poisson(lambda)

y = pd x pa x N etc.

RESPONSE: We have added text in the first paragraph of Methods / Analyses that provides an overview of the hierarchical model and the links between the population and observation sub-models.

Secondly include a complete table of all models fit and any relevant parameters involved in the selection process (e.g. bayes p value), probably in the supplementary material.

RESPONSE: We have described our full model development procedure, including all fitted models and selection criteria in Supplement S2.

Thirdly, the code supplied does not meet modern standards for reproducibility, and it should.
At present JAGS model code is included in supp 2. There is no R code but a wrapper around the model. There is no data provided.

Although the authors state in the declaration to the journal that "all data are fully available without restriction", this does not appear to be the case. The description of where the data may be found points to a series of reports with summaries of the data, not the data themselves. It is not clear where the raw data may be obtained of if these truly are publicly available.

This lack of code and data does not allow others to understand the analysis, let alone reproduce it. I strongly recommend that the raw data be made available if they are not, and that full analysis code be made available and directly linked to it. This may make use of a public code repository such as github, in preference to more code in a word document. Yes this is more work, but it is current good practice and means that anyone should be able to follow your analysis.

RESPONSE: We have cleared the release of all data from the National Park Service, and have provided new links to these data in Supplement S2. We have also previously published all the R code required to process the nutcracker data (cited as [54]).

I cannot stress enough that the methods section is at present very difficult to follow and this must be addressed prior to considering publication. It appears that previous editors and reviewers have been unable to clearly understand what you have done, and this is certainly the case for me. Most readers will not pay nearly as much attention, and so you must make significant efforts to ensure that your work is clear and understandable to even a casual reader.

RESPONSE: We have added text in the first paragraph of Methods / Analyses that provides an overview of the hierarchical model and the links between the population and observation sub-models.

I have a third concern with this study: that the shift from surveying lower to higher elevations later in the year following nutcracker movements is likely to overestimate landscape densities if individuals may be counted at one low elevation site early in the year and other higher elevation sites afterwards. This confounding in sampling means that estimates of N will only be relevant to that precise time, not a general estimate of nutcracker abundance. This point should be addressed in the discussion, and preferrably in future sampling. This is a problem for the generalisability of the conclusions drawn from this sampling.

RESPONSE: We note that our temporal analysis focused on Mount Rainier National Park, where nutcrackers were detected only at high-elevations, so the particular effect that you (rightly) warn of is not relevant to that analysis. However, our other analyses might overestimate nutcracker density for the reason suggested, and we acknowledge this and other limitations to the current temporal aspects of bird sampling design. Our discussion includes, “seasonal variation in resource use requires that we match dates of nutcracker monitoring to the dates of resource use. The timing of standard breeding-bird point-counts might not coincide with the season(s) in which nutcrackers are most frequent or detectable in these parks. Monitoring in the late summer and fall should offer the best opportunity to detect nutcracker use of mature five-needle pine seeds.”

A couple of other minor points:

Abstract: The place name acronyms make the abstract harder to read, not easier. Please don't punish your readers by making them recall four novel acronyms in the abstract.'

RESPONSE: We have removed these acronyms from the abstract.

Table 1 & 2: subscripts a and b are unnecessary - just change "survey period" to "survey months" and state in table label tha count of trees includes live and dead trees.

RESPONSE: We have reduced the number of subscripts using the first change suggested. We retained subscripts that provide key details about the counts of live and dead trees.

L230. Bird surveys?

RESPONSE: We assume this comment is meant to suggest that bird surveys should be addressed in this paragraph. The paragraph has been expanded with a brief description of the model structure, including elements parameterized using data from bird surveys.
